# WBC Image Segmentation Based on Residual Networks and Attentional Mechanisms

**DOI:** 10.1155/2022/1610658

**Published:** 2022-08-31

**Authors:** Jiangping Wu, Xin Zheng, Deyang Liu, Liefu Ai, Pan Tang, Boyang Wang, Yuanzhi Wang

**Affiliations:** ^1^Anqing Normal University, College of Computer and Information, Anqing 246133, Anhui, China; ^2^The University Key Laboratory of Intelligent Perception and Computing of Anhui Province, Anqing Normal University, China, Anqing 246133, Anhui, China; ^3^Jiangxi University of Applied Science, Nanchang 330000, Jiangxi, China

## Abstract

White blood cell (WBC) morphology examination plays a crucial role in diagnosing many diseases. One of the most important steps in WBC morphology analysis is WBC image segmentation, which remains a challenging task. To address the problems of low segmentation accuracy caused by color similarity, uneven brightness, and irregular boundary between WBC regions and the background, a WBC image segmentation network based on U-Net combining residual networks and attention mechanism was proposed. Firstly, the ResNet50 residual block is used to form the main unit of the encoder structure, which helps to overcome the overfitting problem caused by a small number of training samples by improving the network's feature extraction capacity and loading the pretraining weight. Secondly, the SE module is added to the decoder structure to make the model pay more attention to useful features while suppressing useless ones. In addition, atrous convolution is utilized to recover full-resolution feature maps in the decoder structure to increase the receptive field of the convolution layer. Finally, network parameters are optimized using the Adam optimization technique in conjunction with the binary cross-entropy loss function. Experimental results on BCISC and LISC datasets show that the proposed approach has higher segmentation accuracy and robustness.

## 1. Introduction

Cell/colony segmentation and counting play an important role in many biomedical image applications. For example, the differential blood count of optical images is an important index to detect blood diseases such as cancer, anemia, and malaria [1, 2]. For another example, cell colony counting of fluorescence images can be used to measure the effect of radiation, mutagens, carcinogens, chemotherapeutic agents, and other drugs [3]. Yet, manual microscopy is laborious and time-consuming. Besides, the accuracy of the analysis depends on the skills of professionals.

With the rapid development of computer-aided technology, automatic cell/colony segmentation and counting systems could support faster and more reproducible analysis than manual ones. Militello et al. [4] first used the circle Hough transform to detect wells and then extracted colonies based on local adaptive thresholding. Torelli et al. [5] proposed a supervised multithresholding segmentation method aided by a feedback-based watershed algorithm. Sergioli et al. [6] regarded colony segmentation as a binary classification task. After Grey Level Co-occurrence Matrix (GLCM) texture analysis, they used the proposed Quantum-inspired machine learning method to segment colony areas. In summary, this colony segmentation and counting system focus more on counting the number and size of colonies as well as the area covered by colonies. Although the classical learning-based methods rely on hand-crafted features and prior knowledge, they perform well in colony segmentation and counting tasks.

While for a cell segmentation and counting system, e.g., a hemocytometer, requires higher segmentation accuracy and better generalization ability on different individual WBC of the same type and different types. A blood smear image consists mainly of white blood cells (WBCs), red blood cells, and platelets. WBCs are a subset of immune cells that consist of lymphocytes, monocytes, and granulocytes, which are subdivided into neutrophils, basophils, and eosinophils. If there is an unusual number of WBCs of any type, hematologists will perform a further cell morphology examination in detail. Any abnormal WBCs with irregular shapes may trigger the presence of severe diseases [7]. Therefore, in addition to different blood counts, a hemocytometer needs to segment the exact boundaries of different types of WBCs.

With the great success of deep learning over the last decade, convolutional neural networks (CNNs) have been widely applied to cell detection [8, 9], cell segmentation [10–12], cell classification [13, 14], etc. Deep learning can automatically learn the optimized features, which are completely data-driven without hand-crafted features or task-specific knowledge, and thus can segment different types of objects for different segmentation tasks without modifying the model architecture. Despite the need for a large number of pixel-level labeled image datasets for model training, deep learning techniques have outperformed classical learning-based methods for many segmentation tasks.

Lu et al. [11] proposed a WBC segmentation algorithm based on an end-to-end deep CNN network, whose segmentation accuracy and robustness outperform FCN [12] and U-Net [13] networks. Roy et al. [14] used a combination of DeepLabv3+ [15] and ResNet [16] as a feature extraction network to accurately segment WBC images. Similarly, the WBC-Net [17], which combines UNet++ [18] and ResNet, also showed good segmentation performance. To sum up, the method combining ResNet with the existing classical CNNs has many applications in WBC image segmentation.

In addition, the Squeeze-and-excitation (SE) module [19], which is independent of network architecture, has been well applied in the field of medical image segmentation. Qiao and Zulkernine[20] segmented the fetal skull by embedding the SE module into the encoder-decoder CNN structure. To tackle the prostate zonal segmentation task, Rundo et al. [21] proposed a so-called USE-Net, which incorporates the SE module into the U-Net. Li et al. [22] proposed the MSGSE-Net network to accurately segment subcortical brain structures. By using multiscale image context and attention mechanisms, the network focuses on learning discriminative feature representations. Compared with several advanced segmentation methods, this model has better segmentation performance. In short, the presentation of the SE module significantly enhances the representation ability of the CNNs. However, WBC segmentation remains a challenging task due to different lighting conditions, staining techniques, and the inconsistency in cell color, shape, and texture.

Inspired by the application of the ResNet module and the SE module in image segmentation, a new deep learning network is proposed in this paper, which adopts an improved encoder-decoder structure based on U-Net for WBC segmentation. The encoding structure is cascaded with improved residual blocks to improve the feature extraction capability of the network. The SE module was added to the decoding structure to adjust the feature output, and the atrous convolution was integrated to expand the convolution receptive field to obtain multiscale information about images. Experimental results on BCISC [23] and LISC [24] datasets showed that the proposed algorithm can segment WBC images quickly and accurately.

## 2. Related Works

### 2.1. Improving the Residual Module

**Figure 1:**
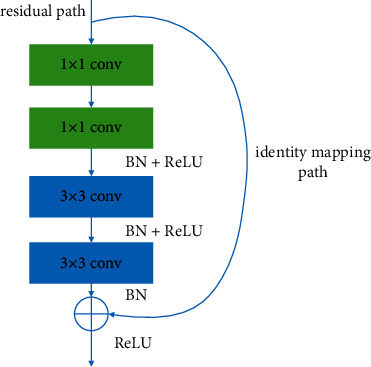
Residual block.

A deep residual retwork is a CNN consisting of multiple residual units. One of the advantages of the ResNet is that it solves the problem of gradient disappearance through the identity mapping path connections. For networks with a large number of hidden layers, after several layers of computation, their gradients will gradually shrink to 0, resulting in the fact that the weights cannot be updated. ResNet solves this problem with a shortcut. The algorithm in this paper uses residual blocks in the encoder to learn more informative WBC features. The structure of residual blocks is shown in Figure 1, which is divided into a residual path and an identity mapping path. The residual path includes 1 × 1 convolution layers, 3 × 3 convolution layers, batch normalization (BN) layers, and ReLU activation layers.

### 2.2. Transfer Learning

Training a deep learning model requires a large amount of training data to prevent overfitting. Unfortunately, pixel-level labeling of medical images is extremely time-consuming work, which can only be done by professionals due to the fact that nonprofessionals may have problems such as mislabeling. In addition to data augmentation, transfer learning [25] can solve the overfitting problem. Transfer learning refers to improving the performance of a new task by transferring the knowledge from related tasks that have already been learned. In this way, pretrained models, which are trained on large-scale image datasets and then fine-tuned on small sample datasets, can be fully utilized. In the proposed network, the pretrained ResNet50 model from the ImageNet dataset [26] is applied, and the parameters are fine-tuned on our datasets. This network not only greatly shortens the training time of the model but also improves its segmentation accuracy.

### 2.3. Attention Mechanism

**Figure 2:**
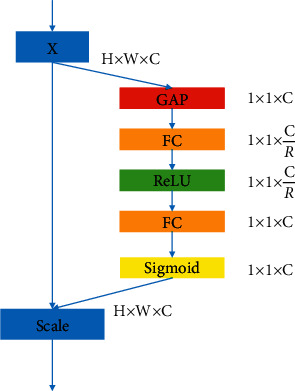
SE inception module.

The SE module [19] is a channel-wise attention mechanism through which the network can selectively learn informative features and suppress useless ones. The schema of the SE module is illustrated in Figure 2. For a given input, it is first passed through the Squeeze operation, i.e., a global average pooling (GAP), to get a channel descriptor. Then, to take advantage of the channel descriptor information aggregated in the Squeeze operation, the dependencies between the channels are captured in the following Excitation operation. This operation first passes through the full connection (FC) layer, and the dimension of feature maps changes from C to C/R. After that, the ReLU activation function is used to make the network more nonlinear, which can better fit the complex correlation between channels. Then, through another FC layer, the feature dimensions are restored, and the normalized weights between 0 and 1 are obtained by the Sigmoid activation function. Finally, the normalized weights are reweighted to each channel feature by a Scale operation.

### 2.4. Atrous Convolution

**Figure 3:**
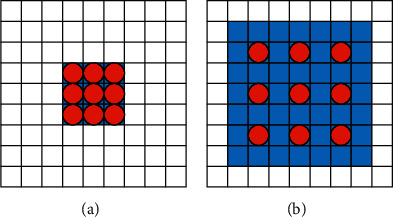
The receptive field under different dilation rate: (a) the dilation rate is 1; (b) the dilation rate is 2.

Atrous convolution, also known as dilated convolution, was proposed to generate high-resolution feature maps. In atrous convolution [27], the receptive field is determined by the dilation rate, which needs to be set manually.  Figure 3 shows the receptive fields under different dilation rates. Assuming that the size of the convolution kernel is 3 × 3 and the dilation rate is 1, the expanded convolution kernel is the same as the original ordinary convolution kernel, as shown in Figure 3(a). When the dilation rate is 2, the size of the convolution kernel remains the same, but the receptive field expands by 7 × 7, as shown in Figure 3(b). The blue area covers the receptive field, and only the red points are involved in the convolution operation. It can be seen that the atrous convolution increases the receptive field while keeping the size of network parameters and output feature graphs unchanged. The receptive field *v* is calculated as follows:

(1) $$\begin{array}{c} v=\left(k+1\right)\times \left(d-1\right)+k, \end{array}$$

where *k* is the kernel size and *d* is the dilation rate.

## 3. The Improved U-Net Integrating Residual Blocks and Attention Mechanisms

The proposed convolutional neural network is improved from the U-Net model. It consists of an encoder and a decoder, which integrates residual blocks and attention mechanisms.

### 3.1. Encoder Structure

**Figure 4:**
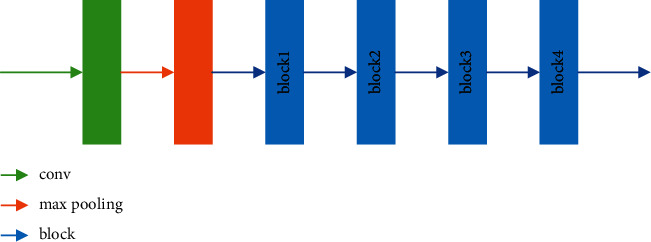
The encoder structure.

In view of the excellent feature extraction ability of ResNet, ResNet50 is modified as the main unit of the encoder. The modified ResNet50 consists of five downsampling units. We remove the average pooling and FC layers, while only the 7 × 7 convolution and max-pooling layer are retained as the first downsampling unit. The remaining four downsampling units are composed of 3, 4, 6, and 3 residual blocks, respectively. The detailed structure of the residual block has been described in detail in Section 2.1, and Table 1 describes the operations of block1, block2, block3, and block4.  Figure 4 shows the structure of the encoder.

### 3.2. Decoder Structure

**Figure 5:**
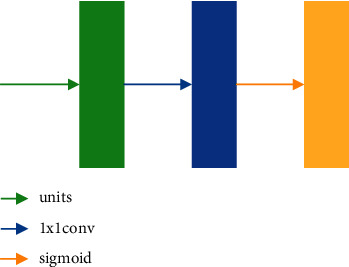
The decoder structure.

The decoder consists of five upsampling units. We upsample the feature maps through bilinear interpolation, and the operations of the first four upsampling units are shown in Table 2, and Figure 5 shows the decoder structure.

In the first four upsampling units, each unit consists of a 1 × 1 convolution layer, a 3 × 3 convolution layer, a BN layer, and a ReLU activation layer, followed by a 3 × 3 atrous convolution layer, a BN layer, a ReLU activation layer, and the SE module. The 1 × 1 convolution layer is used to integrate the feature information between different channels in feature maps. The BN layer can speed up the training speed. The purpose of placing the 3 × 3 atrous convolution layer behind the 3 × 3 convolution layer is that the feature maps first learn the features within a small receptive field through 3 × 3 convolution. Then, the features within a large receptive field are learned by expanding the receptive field through the 3 × 3 atrous convolution. In this way, a multiscale training structure is formed to learn more useful features. Since the SE module has the feature of learning important features and suppressing useless features, it is placed at the end to adjust the feature output.

The last upsampling unit consists of a 1 × 1 convolution layer and the Sigmoid layer. After passing through the 1 × 1 convolution layer, a 64-dimensional feature map is obtained. The weights of the feature map are constrained to 0 to 1 by the Sigmoid layer, and the segmentation result is output.

### 3.3. Improved U-Net Structure

**Figure 6:**
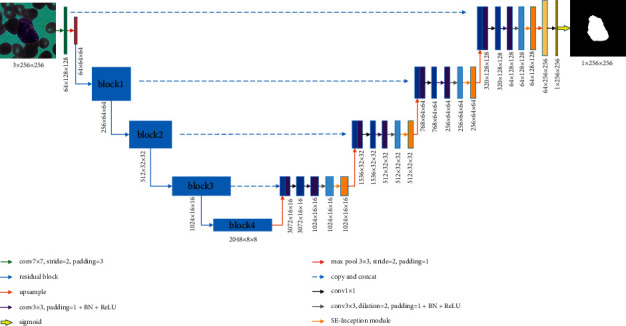
The structure of the proposed network.

The proposed network structure is shown in Figure 6. Take the BCISC dataset as an example, the input is a WBC image with a size of 3 × 256 × 256. A total of five downsampling operations were performed. Firstly, after 7 × 7 convolution and max pooling, a 64 × 64 × 64 feature map was obtained, which is the first downsampling unit. Then, the feature maps of 256 × 64 × 64, 512 × 32 × 32, 1024 × 16 × 16, 2048 × 8 × 8 were obtained through block1, block2, block3, and block4 in turn, which correspond to four downsampling units, respectively. Finally, the image size was reduced from 256 × 256 to 8 × 8. As there is no padding strategy in the original U-Net, which will reduce the image size after the convolution operation, in this network, the padding strategy is added to each convolution layer. So, the feature maps before and after the convolution are of the same size. In addition, to solve the problem of overfitting due to the small amount of data, the encoder is loaded with the weights trained by ResNet50 on the ImageNet dataset. At this point, we have obtained feature maps with sizes of 1024 × 16 × 16, 512 × 32 × 32, 256 × 64 × 64, 64 × 128 × 128, 1 × 256 × 256 at the end of each upsampling unit, respectively, which is the final segmentation image. Since the original U-Net decoder uses deconvolution to restore the size of the feature map, it will cause a checkerboard effect [28]. Hence, the bilinear interpolation method is used to restore the size of the feature maps in the proposed network to make the feature maps smoother.

## 4. Experiment

To test the generalization performance of the proposed network, experiments are carried out on different datasets. Our experiments were performed on a PC equipped with an Intel 2.90 GHz i7 CPU and a NVIDIA Geforce GTX 3090 GPU. The Adam algorithm was used to optimize network parameters in the training process, with a learning rate of 0.0001. Due to the size of the dataset, the batch size was fixed at 8, and the number of epochs was set at 200.

### 4.1. Datasets

Two different WBC image datasets were used to evaluate the segmentation performance. The BCISC dataset consists of 268 single WBC images of 256 × 256, including 51 neutrophils, 54 eosinophils, 56 basophils, 54 monocytes, and 53 lymphocytes. To reduce memory occupancy rate and to facilitate network training, the LISC dataset was cropped into 248 single WBC images with a size of 256 × 256, including 55 neutrophils, 54 eosinophils, 39 basophils, 48 monocytes, and 52 lymphocytes. To fairly compare the proposed model with other methods, we applied data augmentation strategies on both datasets by rotation, flipping, scaling, brightness, and contrast adjustment, and we expanded each dataset to 10000 images. All datasets were randomly divided into the training set, validation set, and test set in a ratio of 8 : 1 : 1.

### 4.2. Evaluation Methods

The commonly used measures for medical image segmentation include Dice Similarity Coefficient [29] (Dice), mean Intersection Over Union [30] (mIOU), Positive Prediction Value (PPV), Sensitivity [31] (SE), and Hausdorff distance [32] (HD). These indexes can be divided into two categories. The first class includes dice, mIOU, and PPV, which are used to evaluate the region's similarity. They are sensitive to the internal regions and range from 0 to 1, with larger values being better. The HD metric belongs to the second class, which is used to evaluate the contour similarity. HD is sensitive to contour fit, and the smaller the value, the better the segmentation performance. These indexes are described as follows:

(2) $$\begin{array}{c} \text{Dice}\left(A,B\right)=\frac{2\left|A\cap B\right|}{\left|A\right|+\left|B\right|},\\ \text{mIOU}\left(A,B\right)=\frac{\left|A\cap B\right|}{\left|A\cup B\right|},\\ \text{PPV}\left(A,B\right)=\frac{\left|A\cap B\right|}{\left|A\right|},\\ \text{SE}\left(A,B\right)=\frac{\left|A\cap B\right|}{\left|B\right|},\\ H\;D\left(A,B\right)=\underset{a\in A}{\max}\left\{\underset{b\in B}{\min}\left\{d\left(a,b\right)\right\}\right\}. \end{array}$$

### 4.3. Loss Function

**Figure 7:**
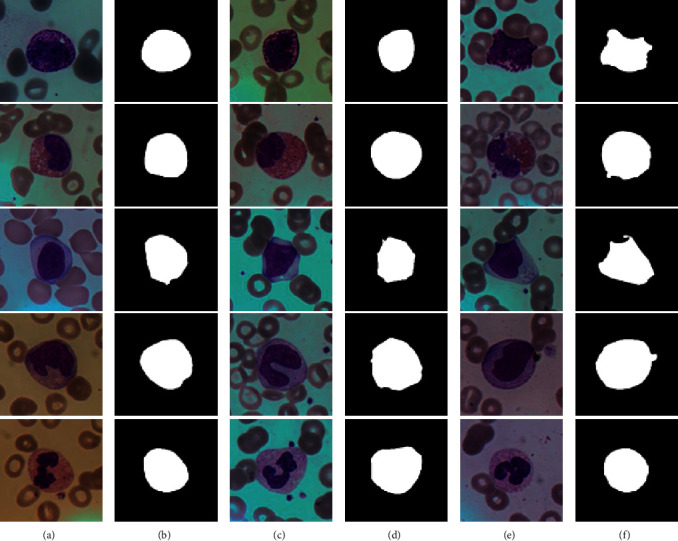
Some sample images of the BCISC dataset. The five rows from top to bottom are basophils, eosinophils, lymphocytes, monocytes, and neutrophils. (a, c, e) The original images; (b, d, f) their corresponding ground truth.

The choice of loss function depends on the characteristics of the datasets.  Figure 7 shows some sample images of the BCISC dataset. The overall image has low brightness, high contrast, and saturation. Besides, the cells have clear boundaries while the shapes are irregular.

It can be observed from the BCISC dataset that, in each of the images, the area of the WBC is similar to that of the background. The segmentation task is to separate WBC regions, and the boundaries of WBCs are clearly defined and easy to distinguish from the background. Therefore, it can be regarded as a general binary classification task. The binary cross-entropy (BCE) loss function is used for network training. Its calculation formula is as follows:

(3) $$\begin{array}{c} \text{loss}=\frac{1}{N}\sum_{i}-\left[y_{i}\log\;p_{i}+\left(1-y_{i}\right)\log\left(1-p_{i}\right)\right], \end{array}$$

where *y*_*i*_ ∈ {0,1} denotes the label of pixel *i*. *y*_*i*_=1 indicates that it belongs to a WBC region; otherwise, it belongs to the background.

**Figure 8:**
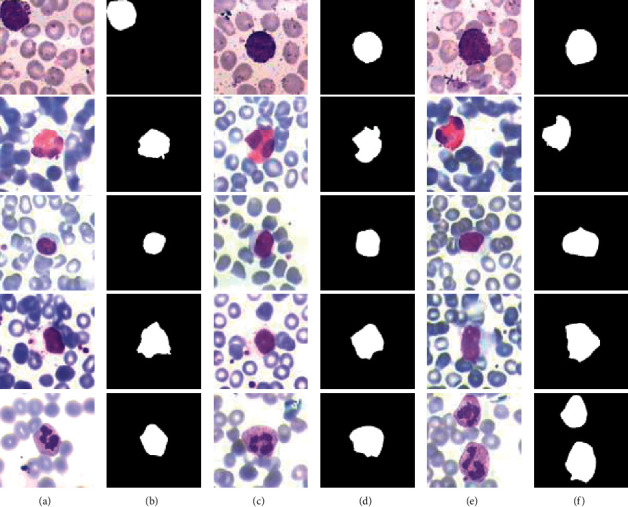
Some sample images of the LISC dataset. The five rows from top to bottom are basophils, eosinophils, lymphocytes, monocytes, and neutrophils. (a, c, e) The original images; (b, d, f) their corresponding ground truth.

Some example images of the LISC dataset are shown in Figure 8. As shown in Figure 8, there is a large color difference between the background and a basophil/neutrophil, which is regularly shaped, so these images can be easily segmented. Although the color difference between eosinophils and the background is large, the shape is irregular and the eosinophils have uneven color, which makes it difficult to segment. It can be indicated that the cytoplasm color of lymphocytes and monocytes is very close to the background color, and the boundaries are difficult to distinguish.

According to the analysis of the LISC datasets, lymphocytes and monocytes are difficult to segment, mainly due to the imbalance between difficult and easy samples. Therefore, Focal Loss [33] was selected as the Loss function of this dataset, where *α*=0.25 and *γ* = 2 were selected. Its calculation formula is as follows:

(4) $$\begin{array}{c} \text{loss}=\left\{\begin{array}{cc} -\alpha {\left(1-p\right)}^{\gamma }\log\left(p\right), & \text{if }y=1,\\ -\left(1-\alpha \right)p^{\gamma }\log\left(1-p\right), & \text{if }y=0, \end{array}\right. \end{array}$$

where *y* is the label of each pixel; *p* is the probability that this pixel is predicted to be positive. Compared with the BCE function, two parameters *α* and *γ* are added in the Focal Loss function to fix imbalance of negative and positive examples and imbalance between difficult and easy examples, respectively.

### 4.4. Data Postprocessing

There are small pieces of the non-WBC area and small holes in the segmentation results. To further remove the small pieces of the non-WBC area and fill the small holes, the segmentation results of all methods were processed by the mathematical morphology.

### 4.5. Evaluation of *R* Value in the SE Module

**Figure 9:**
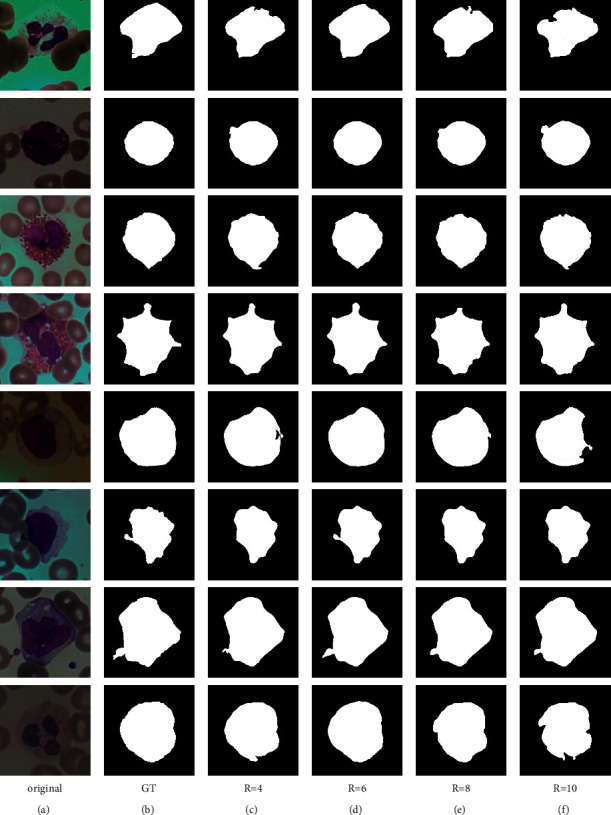
Segmentation results of different (*R*) values. (a) Original. (b) GT. (c) *R* = 4. (d) *R* = 6. (e) *R* = 8. (f) *R* = 10.

In addition to the learning rate, the batch size, and the number of epochs, the *R* value in the SE module is a hyperparameter in the network which needs to be set manually. To obtain better segmentation accuracy, we trained on the BCISC datasets with *R* values set to 4, 6, 8, and 10 respectively in the comparative experiments.  Figure 9 shows the segmentation results for different *R* values, and Table 3 lists the five segmentation measures obtained by applying different *R* values on the BCISC dataset. It can be observed that the proposed method achieves more accurate segmentation results when *R* is 6.

### 4.6. Comparison of Different WBC Segmentation Methods

**Figure 10:**
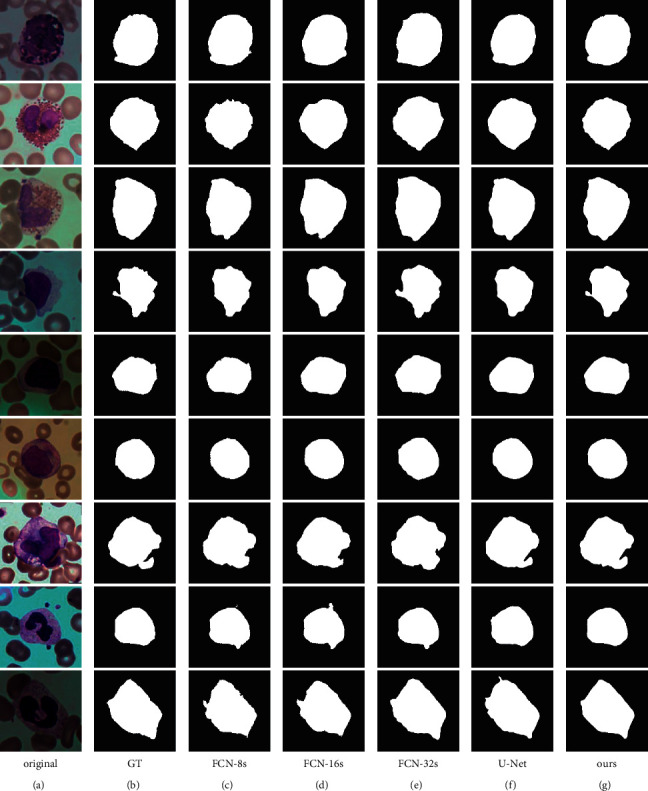
Segmentation results of different methods on the BCISC dataset. (a) Original. (b) GT. (c) FCN-8s. (d) FCN-16s. (e) FCN-32s. (f) U-Net. (g) Ours.

The proposed method was compared with FCN-8s, FCN-16s, FCN-32s, and U-Net on the BCISC dataset. The experimental results are shown in Figure 10, and the quantitative accuracy comparisons are shown in Table 4.

As can be seen from Figure 10, FCN-8s and U-Net suffer from oversegmentation, where the cell contours of their segmentation results are rough and rugged. In addition, the segmentation contours of FCN-16s and FCN-32s are too smooth and do not conform to the ground truth contours. In contrast, the segmentation results of our algorithm are more similar to the ground truth.

Experimental results show that FCN-8s have the highest segmentation accuracy among FCNs. The segmentation accuracy of the proposed method is higher than that of FCN-8s and U-Net. Compared with FCN-8s, the Dice coefficient of our method increases by 1.29%, the mIOU coefficient increases by 2.46%, and the HD decreases by 56.76%. Compared with U-Net, the Dice coefficient is increased by 0.48%, the mIOU is increased by 1.02%, and the HD is decreased by 16.59%. Therefore, the proposed method has higher segmentation accuracy on the BCISC dataset.

**Figure 11:**
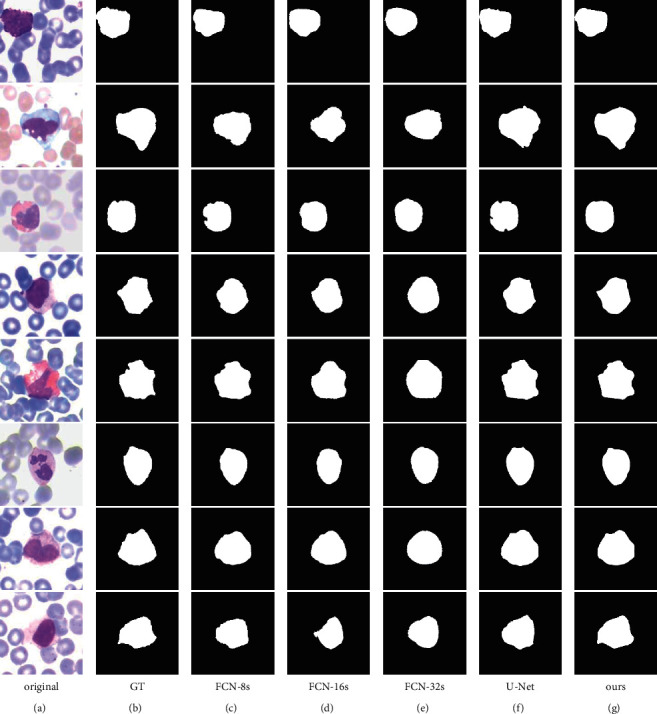
Segmentation results of different methods on the LISC dataset. (a) Original. (b) GT. (c) FCN-8s. (d) FCN-16s. (e) FCN-32s. (f) U-Net. (g) Ours.

In addition, to evaluate the generalization performance of the proposed method, we also conducted experiments on the LISC dataset. The network training parameters were the same as the BCISC datasets. The comparison results are shown in Figure 11, and the evaluation indexes are shown in Table 5.

As can be seen from Figure 11, the oversegmentation problem is more serious for FCN-8s, FCN-16s, and FCN-32s because there is a certain degree of overexposure in the images of the LISC dataset. However, our method can still obtain good segmentation results.

As shown in Table 5, FCN-8s still have the highest segmentation accuracy among FCNs. Compared with FCN-8s, the Dice coefficient and mIOU indexes of our method are improved by 1.95% and 3.24%, respectively, and HD is reduced by 25.38%. Compared with U-Net, the Dice coefficient and mIOU indexes of our method are improved by 0.48% and 0.88%, respectively, and HD is reduced by 11.57%. Therefore, the proposed method has the highest accuracy compared with FCNs and U-Net on the LISC dataset.

## 5. Conclusion

In this paper, we propose a U-Net-based white blood cell image segmentation method integrating residual blocks and attention mechanisms. To solve the problem of insufficient labeled training images, transfer learning of ResNet50 is applied in the encoder of the proposed network, which is used to transfer the information learned from the ImageNet dataset to a small WBC dataset. To make the segmentation results more accurate, the SE module is added to the decoder of the proposed network. Experimental results showed that the proposed method achieves higher accuracy with better robustness to blurred boundaries on different WBC datasets compared with existing representative methods.

In addition to peripheral blood smear image segmentation, the proposed method could be used to segment different kinds of cell images. In future work, we would like to apply the proposed segmentation method to an automated cell/colony counting system.

## Figures and Tables

**Table 1:** The operations for block1, block2, block3, and block4.

Layer name	Block1	Block2	Block3	Block4
Layer	$\left[\begin{array}{c} \text{ conv }1\times 1\text{, }64\;\;\\ \text{ conv }1\times 1\text{, }64\\ \text{ BN}\\ \text{ ReLU}\\ \text{ conv }3\times 3\text{, }64\\ \text{ BN}\\ \text{ ReLU}\\ \text{ conv }1\times 1\text{, }256\\ \text{ BN} \end{array}\right]\times 3$	$\left[\begin{array}{c} \text{ conv }1\times 1\text{, }256\;\\ \text{ conv }1\times 1\text{, }128\\ \text{ BN}\\ \text{ ReLU}\\ \text{ conv }3\times 3\text{, }128\\ \text{ BN}\\ \text{ ReLU}\\ \text{ conv }1\times 1\text{, }512\;\\ \text{ BN} \end{array}\right]\times 4$	$\left[\begin{array}{c} \;\text{conv }1\times 1\text{, }512\;\\ \text{ conv }1\times 1\text{, }256\\ \text{ BN}\\ \text{ ReLU}\\ \text{ conv }3\times 3\text{, }256\\ \text{ BN}\\ \text{ ReLU}\\ \text{ conv }1\times 1\text{, }1024\\ \text{ BN} \end{array}\right]\times 6$	$\left[\begin{array}{c} \text{ conv }1\times 1\text{, }1024\;\\ \text{ conv }1\times 1\text{, }512\\ \text{ BN}\\ \text{ ReLU}\\ \text{ conv }3\times 3\text{, }512\\ \text{ BN}\\ \text{ ReLU}\\ \text{ conv }1\times 1\text{, }2048\\ \text{ BN} \end{array}\right]\times 3$

**Table 2:** The operations of the first four upsampling units.

Upsampling units	Layer
Units	$\left[\begin{array}{c} \text{ conv }1\times 1\;\\ \text{ conv }3\times 3\\ \text{BN}\\ \text{ ReLU}\\ \text{ atrous conv }3\times 3\\ \text{BN}\\ \text{ ReLU}\\ \text{SE}\\ \text{ conv }1\times 1 \end{array}\right]\times 4$

**Table 3:** BCISC dataset segmentation results using different *R*.

*R*	Dice (%)	mIOU (%)	PPV (%)	SE (%)	HD (%)
4	97.90	95.81	98.15	97.67	4.34
6	**98.13**	**96.36**	**98.23**	**98.06**	**3.52**
8	97.90	95.81	98.17	97.65	4.09
10	97.79	95.61	98.10	97.52	4.53

**Table 4:** Comparison of segmentation results of different models on the BCISC dataset.

Model	Dice (%)	mIOU (%)	PPV (%)	SE (%)	HD
FCN-8s	96.84	93.90	97.47	96.38	8.14
FCN-16s	96.79	93.76	97.16	97.53	8.76
FCN-32s	96.55	93.27	93.77	**99.53**	9.02
U-Net	97.65	95.34	97.69	97.65	4.22
Ours	**98.13**	**96.36**	**98.23**	98.06	**3.52**

**Table 5:** Comparison of segmentation results of different models on the LISC dataset.

Model	Dice (%)	mIOU (%)	PPV (%)	SE (%)	HD (%)
FCN-8s	93.36	87.85	97.47	89.90	6.66
FCN-16s	91.97	85.41	94.86	89.80	8.58
FCN-32s	91.04	84.01	**97.75**	85.87	8.84
U-Net	94.83	90.21	94.91	94.95	5.62
Ours	**95.31**	**91.09**	95.87	**94.99**	**4.97**

## Data Availability

The data were obtained from BCISC (https://github.com/fpklipic/BCISC) and LISC (Rezatofighi S H, Soltanian-Zadeh H. Automatic recognition of five types of white blood cells in peripheral blood [J]. Computerized Medical Imaging and Graphics, 2011, 35 (4): 333–343).
